# In Memoriam: Angela Restrepo (1931–2022)

**DOI:** 10.1128/mbio.00627-22

**Published:** 2022-08-16

**Authors:** Daniel R. Matute, Juan G. McEwen

**Affiliations:** a Biology Department, University of North Carolina, Chapel Hill; b Medical Mycology Group, School of Medicine, Universidad de Antioquia, Medellin, Antioquia, Colombia; c Cellular and Molecular Biology Group, Corporación para Investigaciones Biológicas (CIB), Universidad de Antioquia, Medellín, Antioquia, Colombia

**Keywords:** Endemic mycoses, fungi, mycology

## IN MEMORIAM

On February 3, 2022, the medical mycology community lost a true generational talent, an outstanding mycologist who contributed to the understanding of human pathogens from different angles, ranging from microbial genetics to the social and human aspects of infectious disease. Angela Restrepo was born in Medellín, Colombia, in 1931. Over the course of a prolific career, she coauthored over 300 peer-reviewed papers, spanning over 50 years of research. The focus of Restrepo’s career was the biology of fungal pathogens, placing a strong emphasis on the fungus *Paracoccidioides*, the causal agent of paracoccidioidomycosis. Paracoccidioidomycosis is a prevalent mycosis endemic to Central and South America. The disease most commonly affects male rural workers in South America because female hormones suppress the transition of the fungus to a pathogenic form. The findings from Restrepo’s research set up the foundations of the study of endemic mycoses and propelled Latin American mycology onto the scientific map.

The first stage of Angela Restrepo’s career began before her doctoral studies, when she compiled one of the first collections of medically important fungi in South America. She quickly recognized the need for an advanced degree and pursued a Ph.D. at Tulane University, which she completed in 1965. During this time, Restrepo characterized some of the main biological features of *Paracoccidioides* with an emphasis on the biochemical and immunological characterization of its antigens. Notably, Restrepo was instrumental in the development of microbiological techniques to maintain the fungus in synthetic laboratory media, which was a crucial development in the study of its biology. Her approach to studying the life cycle of *Paracoccidioides* was integrative and encompassed ecological, clinical, and immunological approaches, all with the goal of understanding the epidemiology and natural history of fungal pathogens.

In the second stage of her career, one in which we had the privilege to collaborate with her, Restrepo pioneered the use of molecular genetics techniques (including genomics) to understand the extent of genetic diversity in endemic fungal pathogens, including *Paracoccidioides*. These efforts led to developments in molecular diagnostics, the application of molecular epidemiology to understand the prevalence of endemic mycoses, and the application of population genetics to reveal that *Paracoccidioides* is comprised of at least five different species that differ morphologically and in key clinical phenotypes, such as antifungal susceptibility. This research combined classical questions with cutting-edge technology to keep Latin American mycology vibrant and forward-looking.

Angela Restrepo was not only a giant in medical mycology but also a champion of scientific development in Colombia and across the developing world. As she stated on multiple occasions, her scientific legacy, more than any finding, more than any manuscript she wrote, was the people she trained. Over her career, she mentored over 300 students, who in turn seeded the whole field of clinical science across South America. Her direct trainees and those who interacted with her in any capacity recognized her ability to be critical and rigorous, while at the same time fostering confidence. Her legacy of kind rigor will keep looming large for decades to come.[Fig fig1]

**Figure fig1:**
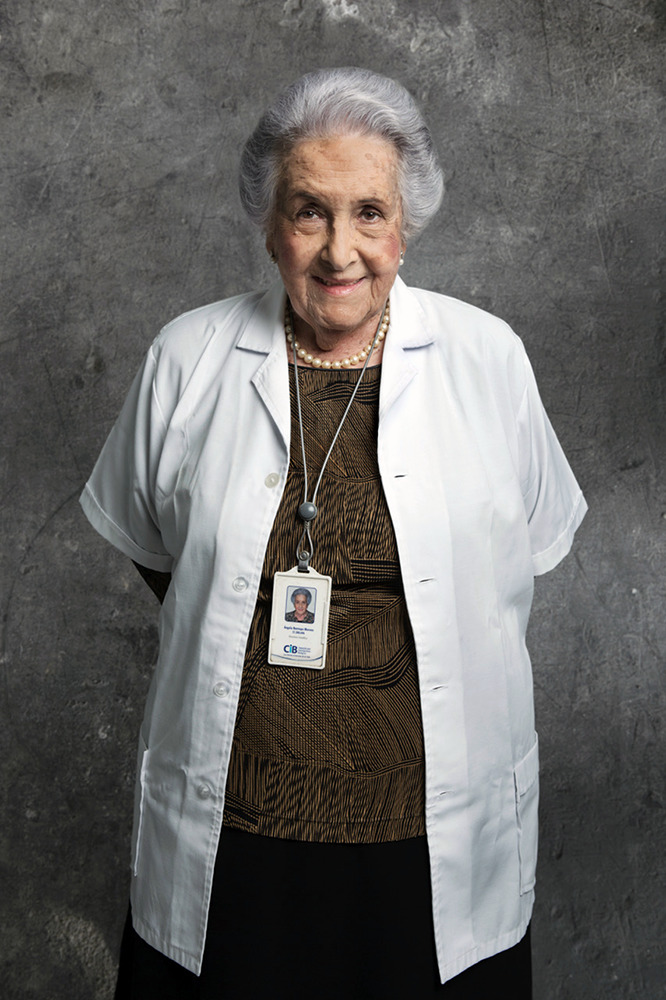
Angela Restrepo in 2014, wearing a badge for the Corporación para Investigaciones Biológicas, the research institute she founded.

